# Perioperative antibiotic prescribing in surgery departments of two private sector hospitals in Madhya Pradesh, India

**DOI:** 10.1186/s13741-019-0121-3

**Published:** 2019-09-10

**Authors:** Anna Machowska, Jonatan Sparrentoft, Shyam Kumar Dhakaita, Cecilia StålsbyLundborg, Megha Sharma

**Affiliations:** 1 Department of Public Health Sciences, Global Health - Health Systems and Policy, Karolinska Institutet, 17177 Stockholm, Sweden; 2 Department of Pharmacology, Ruxmaniben Deepchand Gardi Medical College, Surasa, Ujjain, 456006 India; 3 Department of Surgery, Ruxmaniben Deepchand Gardi Medical College, Surasa, Ujjain, 456006 India

**Keywords:** Perioperative antibiotic prescribing, Non-infectious surgery indications, Urogenital surgery, Abdominal surgery, Empirical prescribing

## Abstract

**Background:**

Single-dose perioperative antibiotic prophylaxis (PAP) is recommended for clean, non-infectious surgeries to prevent surgical site infections. However, the common practice of unindicated use and prolonged use of antibiotics contributes to the development and spread of antibiotic resistance (ABR). The present study explores the perioperative use of antibiotics among inpatients with surgical indications at surgery departments of a teaching (TH) and a non-teaching (NTH) tertiary care hospital in Madhya Pradesh, India.

**Methods:**

Data was collected manually for all inpatients for 3 years (April 2008–August 2011). Patients with non-infectious surgical indications were selected for detailed analysis at the diagnosis group level.

**Results:**

Out of 12,434 enrolled inpatients (TH 6171 and NTH 6263), the majority (> 85%) received antibiotics. None of the inpatients received the recommended single-dose PAP. The average duration of antibiotic treatment was significantly longer at the TH compared to the NTH (9.5 vs 4.4 days, *p* < 0.001). Based on the study aim, 5984 patients were classified in four diagnosis groups: upper or lower urinary tract surgery indications (UUTSI and LUTSI), and routine or emergency abdominal surgery indications (RASI and EASI). In both hospitals, quinolones were the most prescribed antibiotics for UUTSI (TH 70%, NTH 37%) and LUTSI (TH 70%, NTH 61%) antibiotic. In the TH, aminoglycosides (TH 32%) were commonly prescribed for RASI and imidazole derivatives (75%) for EASI. In the NTH, cephalosporins (39%) and imidazole derivatives (56%) were the most prescribed in RASI and EASI, respectively.

**Conclusions and recommendations:**

High prescribing of antibiotics in all four selected diagnoses groups was observed at both hospitals. In spite of the recommended single-dose PAP, antibiotics were mainly prescribed for longer durations. The unrecommended use of antibiotics is a risk factor for the development of AMR. Improving the quality of antibiotic prescribing by a stewardship program focusing on the development and implementation of local prescribing guidelines is needed.

## Background

Antibiotic resistance (ABR) is a definite result of antibiotic use and a condition when common infections or minor injuries can turn into life-threatening situations. Therefore, ABR is one of the major challenges to public health, globally (Gagliotti et al. 2011). Unavailability or poor implementation of antibiotic prescribing guidelines and constrained resources are major contributors to the development and spread of ABR. Prescribing antibiotics for a specific indication according to the local prescribing guidelines enhances effectiveness of the treatment. Development of local prescribing guidelines based on surveillance of antibiotic prescribing and resistance patterns is recommended. Acceptance of the guidelines by the prescribers is essential to assure the compliance of the guidelines. Various strategies have been applied globally, to improve prescribing and compliance, such as computerized registers for the surveillance and follow-up of the prescriptions and offering financial incentives to the prescribers (Gould 2002; Bou-Antoun et al. 2018). However, resource-constrained healthcare facilities of low- and middle-income countries (LMICs) cannot follow most of these strategies and depend on the use of human resources. Manual collection of antibiotic prescribing data for a long time period even with the help of available World Health Organization (WHO) tools is an expensive, time-consuming, and cumbersome process (World Health Organization 2017a).

India is an infection prone country wherein 2011, 30% of all hospital mortalities were reported due to infectious diseases (Ganguly 2011; Census of India 2011). Healthcare facilities are the main source of healthcare-associated infections (HAIs); therefore, high use of antibiotics is anticipated at these settings. HAIs increase morbidity rates, healthcare costs, and mortalities (Chandy et al. 2014; Shojania et al. 2001). In India, 93% of all healthcare facilities belong to the private healthcare sector. Thus, this sector plays a significant role to provide healthcare and medical services and also to the overall increase of antibiotic consumption and resistance in the country (P C. Healthcare in India 2009; Deshpande et al. 2004; World Health Organization 2009). Despite this, little is known about antibiotic prescribing patterns in high infection risk units of the facilities (Sharma et al. 2012). The patients undergoing surgery are at high relative risk of a most common form of HAIs, i.e., the surgical site infections (SSIs) (World Health Organization 2011; Alp et al. 2014). Prescription of antibiotics as perioperative antibiotic prophylaxis (PAP) is recommended to prevent SSIs. PAP is the limited number of antibiotic doses administered along with preoperative preparation, during or after surgeries, in an aseptic condition and postoperative wound care in order to prevent the suspected SSIs (Hohmann et al. 2012). PAPs are recommended to discontinue after 24 h of surgery, in the absence of a known infection (Crader and Bhimji 2018). Thus, analysis of the constituents of the PAP and assessment of prescribing of antibiotics based on the indications is crucial.

The overall use of antibiotics is at the rapid rise in India where beta-lactam antibiotics and quinolones are the most commonly used classes (World Health Organization 2011; World Health Organization 2012). Although least investigated, yet extensive PAP practices in India could be presumed in view of reports of high antibiotic prescribing (Sharma et al. 2012; Van Boeckel et al. 2014; Alvarez-Uria et al. 2014). Studies conducted at surgery departments of two Indian healthcare settings presented that nearly 90% of the inpatients were prescribed antibiotics and SSI rate was 5% in one of the settings (Sharma et al. 2012; Pathak et al. 2014). In a study conducted at the study setting between 2010 and 2013, the SSI rate ranged between 5 and 6.5% (Lindsjo et al. 2015). Despite a paucity of antibiotic prescribing surveillance studies from India, rapid development and spread of ABR could be anticipated due to extensive use of antibiotics. This stimulates to conduct a study on antibiotic prescribing in high infection risk departments such as surgery department at Indian healthcare facilities (Hawkey 2008). The resistance situation is worrisome as previous studies from the settings based on 716 isolates from 2568 patient showed that 69% of *E.coli* and 41% of *Klebsiella pneumoniae* isolates were ESBL producers. The isolates had high resistance to fluoroquinolones and beta-lactams except for imipenem and piperacillin-tazobactam. Methicillin-resistant *Staphylococcus aureus* (MRSA) showed high resistance to ciprofloxacin, co-trimoxazole, and levofloxacin (Pathak et al. 2012).

Thus, the aim of the present study is to explore the perioperative use of antibiotics among inpatients with the most common surgical indications at surgery departments of a teaching (TH) and a non-teaching (NTH) tertiary care hospital in Ujjain district of Madhya Pradesh, India.

## Methods

### Study setting

Surgery departments of two private, tertiary care hospitals run by a trust in Ujjain district, Madhya Pradesh, India, were included in the study. The Chandrikaben Rashmikant Gardi Hospital, a rural-based teaching hospital (TH), is located in the village Surasa and has 5 operation theaters and 130 beds at the surgery departments. The TH is run on a charity basis where healthcare services and medications are provided free of cost to all in- and outpatients. All medicines are procured by the management. The Ujjain Charitable Trust Hospital, the urban-based non-teaching hospital (NTH), is located in the city of Ujjain and has 36 beds at the surgery department and 5 operation theaters. At the NTH, the healthcare services are available at a subsidized rate yet patients have to pay for all prescribed medicines out-of-pocket.

### Data collection and analysis

This was a cross-sectional observational study with data collected over 3 years. Data was recorded manually for all patients admitted to the surgery department using a customized data collection tool. The nursing staff was trained for data collection (Sharma et al. 2012). Information such as demographic details; hospitalization dates; all diagnoses (indications), written by the treating surgeon; and details of prescribed antibiotics were recorded from the patients’ file. All diagnoses were coded according to the International Statistical Classification of Diseases and Related Health Problems-Tenth Revision (ICD-10) (World Health Organization 2010). Specifically trained persons fill in the generic names and Anatomical Therapeutic Chemical classification (ATC) codes of the antibiotics that were prescribed by brand names (World Health Organization 2017a). The ATC codes of new FDCs that were not recognized by the WHO were assigned by the authors in agreement with WHOCC in Norway (Sharma et al. 2012). Defined daily doses (DDD) prescribed per day were calculated for prescribed antibiotics (World Health Organization 2017a). Antibiotic prescriptions were analyzed for the adherence to the WHO List of Essential Medicines (WHOLEM) (World Health Organization 2017b) and the National List of Essential Medicines of India (NLEMI) (Directorate General of Health Services MoHaFW, Government of India 2011). The analysis was conducted at the substance level of the ATC for selected diagnoses groups of the patients (World Health Organization 2017a).

**Fig. 1:**
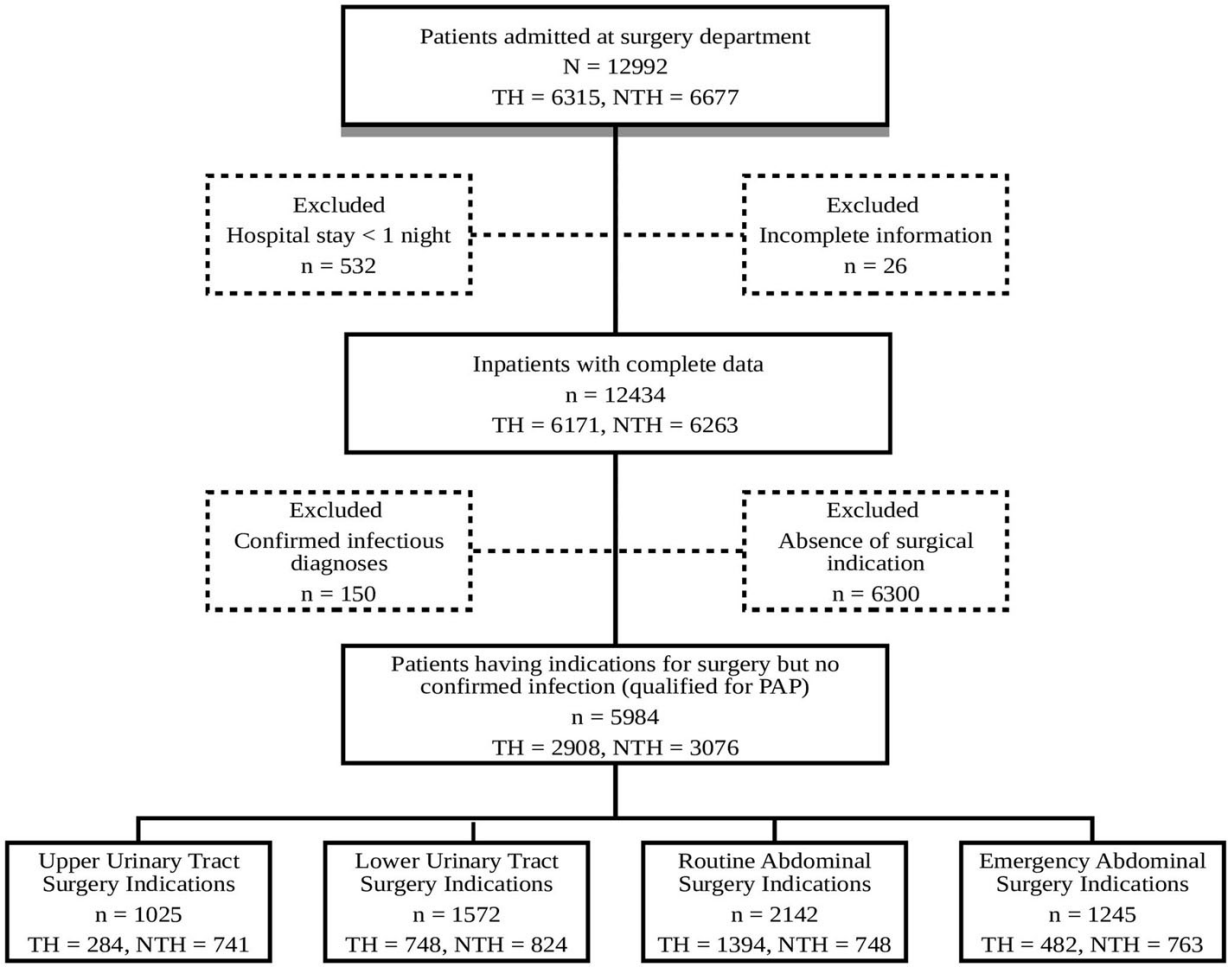
Diagrammatic representation of the analysis process of inpatients data from the surgery departments of two hospitals. PAP, perioperative antibiotic prophylaxis; TH, teaching hospital; NTH, non-teaching hospital

The surgeries of the urinary tract and abdominal surgeries were most common in the settings. Patients who underwent these most commonly performed surgical procedures but had no suspected bacterial infections were selected for detailed analysis. In total, 5984 who had urinary tract and abdominal surgeries were selected and grouped in four diagnoses groups, namely upper urinary tract surgery indications (UUTSI), lower urinary tract surgery indications (LUTSI), routine abdominal surgery indications (RASI), and emergency abdominal surgery indications (EASI). As the patients appearing for emergency abdominal surgery are unique in terms of varied risk factors than the routine surgeries, thus, they were grouped and analyzed separately. All patients who had simultaneous diagnoses in the ICD-10 group A (721), i.e., bacterial infections; ICD-10 group B (105), i.e., other infections group; and urinary tract infections (UTIs) from ICD-10 group N were excluded from the analysis. No national PAP guidelines are available in India; therefore, international guidelines were used as a reference in the present communication (World Health Organization 2016). The selection process for detailed analysis is presented as a flowchart in Fig. 1, according to the STROBE guidelines (Strengthening the Reporting of Observational studies in Epidemiology-STROBE statement n.d.).

### Inclusion and exclusion criteria

Overall, 12,992 patients were admitted to the surgery departments during 2008–2011 (Fig. 1). Patients who had stayed for at least one night in either hospital and had complete data were included for the analysis. Patients who had not had a surgical indication and/or who had confirmed microbiology culture test reports for a bacterial infection were excluded from further analysis.

### Statistical analysis

Excel and STATA software version 15.0 (Stata Corp., College Station, Texas, USA) were utilized for the statistical analysis. For categorical variables, frequency and percentages were calculated. Sum, mean, and standard deviation were calculated for continuous values. The independent sample *t* test was used to compare continuous variables and chi-square tests to compare categorical variables. The *p* value < 0.001 was considered significant. The low *p* value was selected as a large number of variables were analyzed which might lead to a large risk of type I errors (false-positive results).

## Results

### Antibiotic prescribing at the department level

Among 12,992 enrolled inpatients, 6315 were admitted at the rural (TH) and 6677 at the urban hospital (NTH, Fig. 1). Of these, 12,434 inpatients fulfilled inclusion criteria and were included in the study: 6171 (98%) patients at the TH and 6263 (94%) at the NTH. Antibiotics were prescribed to 88% inpatients in the TH and 86% in the NTH. The length of hospital stay ranged between 1 and 129 days at TH, and 1 and 56 days at the NTH; antibiotic treatment ranged between 1 and 110 days, and 1 and 44 days at the TH and NTH, respectively (Table 1). Both the average length of hospital stay (9.9 vs 4.6 days) and the average length of antibiotic treatment (9.5 vs 4.4 days) were significantly higher in the TH compared to the NTH.

**Table 1:** Inpatients’ characteristics at surgery departments of the TH and NTH in Madhya Pradesh, India

	All patients		UUTSI		LUTSI		RASI		EASI	
	TH, *N* = 6171	NTH, *N* = 6263	TH, *N* = 284	NTH, *N* = 741	TH, *N* = 748	NTH, *N* = 824	TH, *N* = 1394	NTH, *N* = 748	TH, *N* = 482	NTH, *N* = 763
	*n* (%)	*n* (%)	*n* (%)	*n* (%)	*n* (%)	*n* (%)	*n* (%)	*n* (%)	*n* (%)	*n* (%)
Age in years										
0–17	*677 (11)* ^a^	377 (6)	21 (7)	36 (5)	*45 (6)* ^a^	15 (2)	*139 (10)* ^a^	26 (3)	72 (15)	91 (12)
18–39	2018 (33)	*2757 (44)* ^a^	159 (56)	425 (57)	69 (9)	119 (14)	320 (23)	*281 (38)* ^a^	239 (50)	419 (55)
40–59	1773 (29)	1689 (27)	78 (27)	197 (27)	150 (20)	148 (18)	453 (32)	222 (30)	108 (22)	188 (25)
≥ 60	*1703 (28)* ^a^	1440 (23)	26 (9)	83 (11)	484 (65)	542 (66)	482 (35)	219 (29)	63 (13)	65 (8)
Male	*4601 (75)* ^a^	4384 (70)	218 (77)	546 (74)	719 (96)	812 (99)	*1182 (85)* ^a^	532 (71)	347 (72)	610 (80)
Female	1570 (25)	*1879 (30)* ^a^	66 (23)	195 (26)	29 (4)	12 (1)	212 (15)	*216 (29)* ^a^	135 (28)	153 (20)
Patients prescribed AB	5430 (88)	5402 (86)	251 (88)	624 (84)	748 (100)	823 (100)	*1393 (100)* ^a^	628 (84)	*482 (100)* ^a^	719 (94)
Total number of AB prescriptions (Np)	12,213	9598	563	1055	1624	1453	2767	1240	1435	1679
Prescriptions adherent to WHOLEM, Np (percentage of total nr. of prescriptions)	*8039 (66)* ^a^	4019 (42)	*309 (55)* ^a^	346 (33)	*753 (46)* ^a^	346 (24)	*1736 (63)* ^a^	510 (41)	*1054 (73)* ^a^	790 (47)
Prescriptions adherent to NLEMI, Np (percentage of total nr. of prescriptions)	*9119 (75)* ^a^	5487 (57)	342 (61)	592 (56)	*880 (54)* ^a^	592 (41)	*2025 (73)* ^a^	734 (59)	*1173 (82)* ^a^	984 (59)
Average length of hospital stay, days [SD]	*9.9 [10.0]* ^a^	4.6 [3.9]	*9.1 [7.8]* ^a^	3.8 [3.2]	*13.0 [11.3]* ^a^	5.0 [3.2]	*9.8 [7.2]* ^a^	5.1 [4.0]	*11.1 [9.5]* ^a^	6.3 [5.3]
Average length of AB treatment, days [SD]	*9.5 [7.6]* ^a^	4.4 [3.2]	*8.6 [6.9]* ^a^	3.6 [2.6]	*11.5 [9.0]* ^a^	4.9 [3.0]	*7.8 [5.3]* ^a^	4.4 [3.4]	*9.0 [7.4]* ^a^	5.5 [4.0]
AB prescribed for 1 day	36 (1)	221 (7)	7 (2)	96 (13)	6 (1)	44 (5)	14 (1)	41 (5)	9 (2)	40 (5)
DDD/100 patient days	72.5	*110.5* ^a^	89.5	*112.3* ^a^	84.6	*102.1* ^a^	78.3	*116.1* ^a^	74.9	*106.0* ^a^
Total nr. of AB doses administered	65,288	28,600	2759	2950	9415	4733	13,890	4060	6270	7980
Total nr. of AB doses prescribed using generic names	*16,322 (25)* ^a^	1716 (6)	*607 (22)* ^a^	118 (4)	*2542 (27)* ^a^	142 (3)	*4584 (33)* ^a^	203 (5)	*1693 (27)* ^a^	399 (5)
Total nr. of AB doses administered parenterally	34,613 (53)	*19,639 (79)* ^a^	1335 (41)	*2245 (76)* ^a^	3672 (39)	*2603 (55)* ^a^	8195 (59)	*3370 (83)* ^a^	4582 (75)	*7339 (92)* ^a^

Variables are presented either with percentages or standard deviations in parenthesis. Percentages were rounded off to integers, and standard deviations were rounded off to one decimal. Percentages are presented in parenthesis, and standard deviations in square brackets. *Abbreviations*: *AB* antibiotic, *DDD* defined daily dose, *EASI* emergency abdominal surgery indications, *Np* total number of antibiotic prescriptions, *nr.* number, *LUTSI* lower urinary tract surgery indications, *NLEMI* National List of Essential Medicines of India, *NTH* non-teaching hospital, *RASI* routine abdominal surgery indications, *SD* standard deviation, *TH* teaching hospital, *UUTSI* upper urinary tract surgery indications, *WHOLEM* WHO List of Essential Medicines

^a^ Significantly larger *p*-values

In the NTH, the DDDs/100 patient days was significantly higher (110.5 vs 72.5, *p* < 0.001) and a larger proportion of the antibiotics were administered by parenteral route (79% vs 53%, *p* < 0.001) than in the TH. Antibiotics were advised after hospital discharge to 1349 patients 1359 (22%) in the TH and to 3136 (50%) patients in the NTH. Overall, significantly larger proportion of antibiotics were prescribed by generic names (25% vs 6%, *p* < 0.001) and prescriptions were more adherent to WHOLEM (66% vs 42%, *p* < 0.001) and NLEMI (75% vs 57%, *p* < 0.001) in the TH compared to NTH (Table 1).

### Diagnosis panorama

Gastrointestinal diseases (ICD-10: K, 33%), urogenital diseases (ICD-10: N, 28%), and skin diseases (ICD-10: L, 9%) were the most common diagnoses groups, sorted by the first level of ICD-10 classification system, at surgery departments of the TH (*n* = 12,434) (World Health Organization 2010). Whereas in the NTH, urogenital diseases (ICD-10: N, 38%), gastrointestinal diseases (ICD-10: K, 26 %), and symptoms without specific diagnoses (ICD-10: R, 12 %) were most commonly recorded.

Of the total 12,434 inpatients, 150 patients had either microbiology reports to confirm the presence of a bacterial infection or a clear clinical indication for the presence of bacterial infection. A total of 6300 inpatients were discharged from the departments without undergoing any surgery leaving 5984 inpatients for further detailed analysis (Fig. 1).

These 5984 inpatients were divided into four diagnoses groups as described in the “Methods” section (UUTSI, LUTSI, RASI, and EASI). Overall, ventral or inguinal hernias (*n* = 1277), benign prostate hyperplasia (*n* = 1080), kidney or ureter stones (*n* = 1014), appendicitis (*n* = 727), and intestinal obstructions (*n* = 334) were the most common diagnoses. Table 2 presents the list of most common diagnoses among the selected inpatients.

**Table 2:** Classification of surgery inpatients in four selected diagnoses groups, based on their diagnoses

Diagnosis groups (ICD-10 codes)	TH, *n* = 2908 (100)	NTH, *n* = 3076 (100)
	*n* (%) [%]	*n* (%) [%]
Upper urinary tract surgery indications (UUTSI)	284 (10) [100]	*741(24) [100]* ^a^
Kidney/ureter calculi (N20)	280 [99]	*734 [99]* ^a^
Other	4 [32]	7 [1]
Lower urinary tract surgery indications (LUTSI)	748 (26) [100]	824 (27) [100]
Benign prostate hyperplasia (N40)	522 [70]	558 [68]
Urethral/bladder calculi (N21)	89 [12]	*142 [17]* ^a^
Urethral stricture (N35)	85 [11]	66 [8]
Prostate cancer (C61)	29 [4]	30 [4]
Bladder cancer (C67)	10 [1]	10 [1]
Urethral disease* (N36)	9 [1]	10 [1]
Other	4 [< 1]	8 [< 1]
Routine abdominal surgery indications (RASI)	*1394 (48) [100]* ^a^	748 (24) [100]
Hernia (K40-K46)	*965 [69]* ^a^	312 [42]
Other gastric/duodenal disease (K31)	113 [8]	151 [20]
Biliary calculi (K80)	73 [5]	110 [15]
Anal fissure/fistula (K60)	*103 [7]* ^a^	30 [4]
Malignancy in GI-tract (C15-C26)	78 [6]	51 [7]
Cholecystitis (K81)	30 [2]	43 [6]
Anal abscess (K61)	10 [< 1]	28 [4]
Other disease in anal region** (K62)	10 [< 1]	8 [1]
Colon polyposis (K63.2)	9 [< 1]	5 [< 1]
Other	3 [< 1]	10 [< 1]
Emergency abdominal surgery indications (EASI)	482 (17) [100]	*763 (25) [100]* ^a^
Appendicitis (K35-K37)	268 [56]	*459 [60]* ^a^
Intestinal obstruction (K56)	132 [27]	202 [27]
Peritonitis (K56)	80 [17]	99 [13]
Other	2 [< 1]	3 [< 1]

Numbers of patients with percentages rounded off to the nearest whole number. *Abbreviations*: *TH* teaching hospital, *ns p* value value not significant, *NTH* non-teaching hospital

^*^Urethral disease includes fistulas, diverticulosis, carbuncles, and prolepses of the urethra

^**^Disease in the anal/rectal region includes polyps, stenosis, wounds, bleedings, and prolapses in the anal/rectal region

^a^ Significantly larger *p*-values

### Antibiotic prescribing in four selected surgical diagnoses groups

The average length of hospital stay was significantly longer in the TH (9.1 to 13.0 days) across all selected diagnoses groups compared to the NTH (3.8 to 6.3 days) (Table 1). The inpatients in the TH also had a significantly longer duration of antibiotic treatment in all diagnoses groups with an average between 7.8 and 11.5 days, while in the NTH, it ranged between 3.6 and 5.5 days. Exclusive PAP was not observed in any of the cases in both hospitals. However, in the TH and NTH, 1% and 7% of inpatients, respectively, were prescribed antibiotics for 1 day. A significantly higher proportion of inpatients in both the RASI (100% vs 84%) and the EASI groups (100% vs 94%) were prescribed antibiotic treatment in the TH compared to the NTH, respectively. However, there was no significant difference between the proportions of patients being prescribed antibiotics for the UUTSI and the LUTSI. In general, the prescriptions from the TH adhered significantly more to the available prescribing guidelines (NLEMI and WHOELM) in all diagnosis groups compared to the NTH (Table 1).

**Fig. 2:**
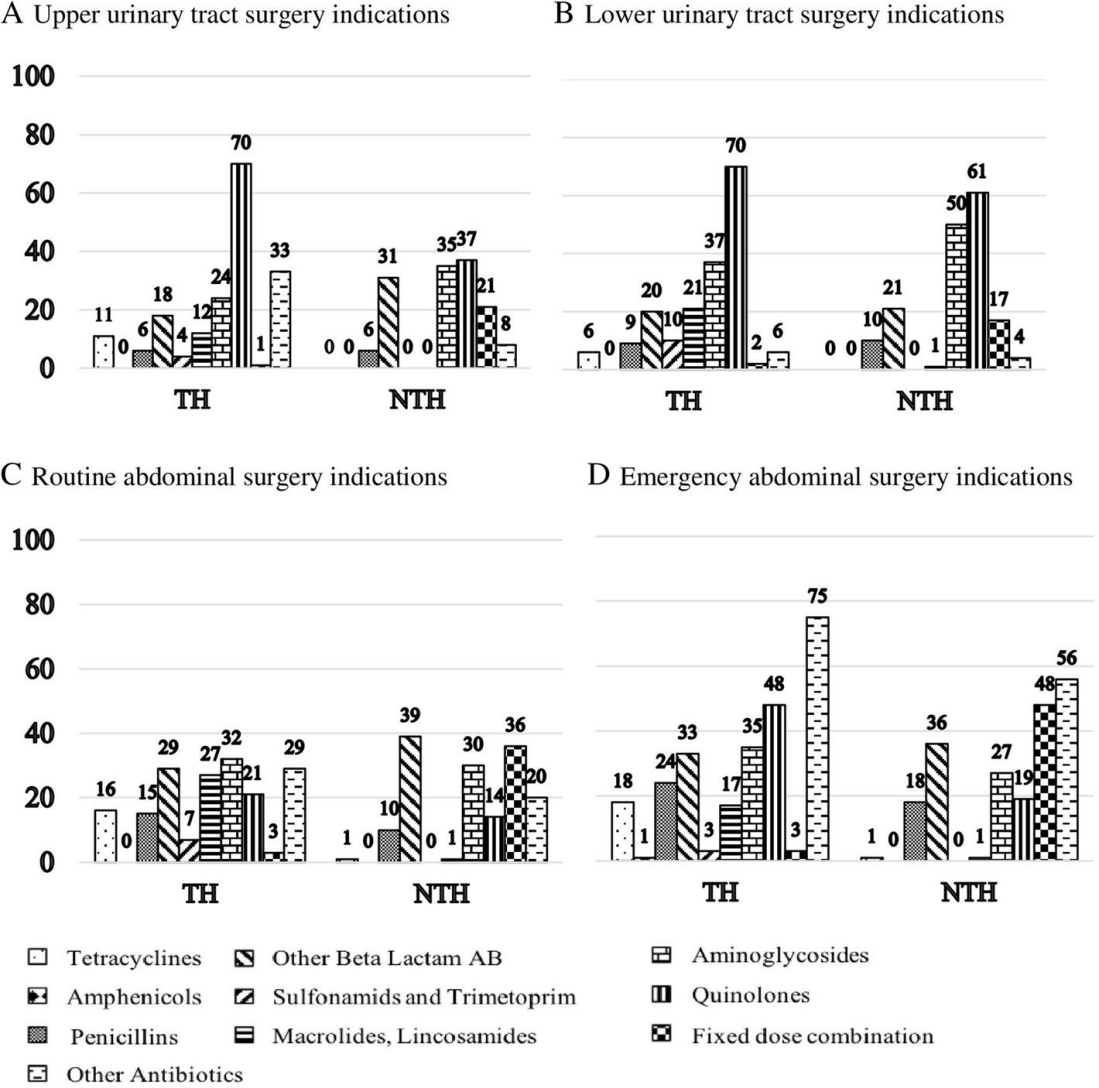
Proportions of inpatients prescribed antibiotics in selected non-infectious surgical diagnoses groups (**a** to **d**) in two study hospitals. Numbers in graphs indicate percentages of patients and are rounded off to nearest integers. AB, antibiotics; *N*, total number of prescriptions in the diagnosis group; n, number of prescriptions for the third-level ATC pharmacological sub-group; TH, teaching hospital; NTH, non-teaching hospital

Fixed-dose combinations (FDCs) of antibiotics and third-generation cephalosporins were prescribed to a larger extent in all diagnosis groups in the NTH (Table 3). With regard to the proportion of prescribed antibiotics for the selected diagnoses groups, quinolones appeared to be the highest prescribed therapeutic sub-group in the UUTSI and LUTSI groups in both hospitals (Table 3).With respect to the number of patients, most of the inpatients in the LUTSI group received quinolones (TH 70% and NTH 61%) and aminoglycosides (TH 37% and NTH 50%). In RASI group in the TH, most of the inpatients received aminoglycosides (32%), other beta-lactam antibiotics (cephalosporins, 29%), and imidazole derivatives (29%), whereas in the NTH, other beta-lactam antibiotics (cephalosporins, 39%) and FCDs (36%) were the most prescribed. The highest proportion of patients in the EASI received imidazole derivatives (TH 75% and 56% NTH), FDCs (NTH 48%), and quinolones (TH 48%; Fig. 2).

**Table 3:** Antibiotic prescribing patterns in four surgical diagnoses groups at surgery departments of the TH and NTH

	UUTSI		LUTSI		RASI		EASI	
	TH, *N* = 563	NTH, *N* = 1055	TH, *N* = 1624	NTH, *N* = 1453	TH, *N* = 2767	NTH, *N* = 1240	TH, *N* = 1435	NTH, *N* = 1679
	*n* (%)	*n* (%)	*n* (%)	*n* (%)	*n* (%)	*n* (%)	*n* (%)	*n* (%)
Tetracyclines (J01A), *n* (% of *N*)	32 (6)	2 (< 1)	50 (3)	0	233 (8)	6 (< 1)	98 (7)	2 (< 1)
Amphenicols (J01B), *n* (% of *N*)	0	0	0	0	0	0	1	0
Penicillins (J01C), *n* (% of *N*)	17 (3)	42 (4)	77 (5)	80 (6)	219 (8)	80 (6)	131 (9)	147 (9)
Penicillins with extended spectrum (J01CA), *x* (% of *n*)	3 (18)	2 (5)	5 (6)	4 (5)	21 (10)	16 (20)	0	10 (7)
Penicillins combinations including enzyme inhibitors (J01CR), *x* (% of *n*)	14 (82)	40 (95)	72 (94)	76 (95)	198 (90)	64 (80)	131 (100)	137 (93)
Other beta-lactam antibiotics (J01D), *n* (% of *N*)	70 (12)	239 (23)	220 (14)	191 (13)	535 (19)	332 (27)	218 (15)	296 (18)
First-generation cephalosporins J01DB, *x* (% of *n*)	19 (27)	7 (3)	49 (22)	1 (< 1)	149 (28)	17 (5)	50 (23)	11 (4)
Second-generation cephalosporins J01 DC, *x* (% of *n*)	0	9 (4)	13 (6)	24 (13)	23 (4)	41 (12)	5 (2)	29 (10)
Third-generation cephalosporins J01DD, *x* (% of *n*)	51 (72)	222 (93)	158 (72)	166 (87)	363 (68)	272 (82)	162 (74)	254 (86)
Fourth-generation cephalosporins J01DE, *x* (% of *n*)	0	1 (< 1)	0	0	0	1 (< 1)	0	0
Carbapenems J01DH, *x* (% of *n*)	0	0	0	0	0	1 (< 1)	1 (< 1)	2 (< 1)
Sulfonamids and trimethoprim (J01E), *n* (% of *N*)	11 (2)	0	85 (5)	0	100 (4)	0	16 (1)	0
Macrolids and lincosamids (J01F), *n* (% of *N*)	38 (7)	0	180 (11)	2 (< 1)	405 (15)	3 (< 1)	94 (7)	8 (< 1)
Macrolids (J01FA), *x* (% of *n*)	0	0	4 (2)	0	3 (< 1)	0	1 (1)	0
Lincosamids (J01FF), *x* (% of *n*)	38 (100)	0	176 (98)	2 (100)	402 (99)	3	93 (99)	8 (100)
Aminoglycosides (J01G), *n* (% of *N*)	76 (13)	269 (26)	328 (20)	449 (31)	486 (18)	250 (20)	189 (13)	225(13)
Quinolones (J01 M), *n* (% of *N*)	216 (38)	281 (27)	609 (38)	543 (37)	315 (11)	114 (9)	263 (18)	145 (9)
Fixed-dose combinations (J01R), *n* (% of *N*)	3 (1)	160 (15)	22 (1)	155 (11)	37 (1)	298 (24)	17 (1)	399 (24)
Quinolones and imidazole derivatives*, *x* (% of *n*)	0	2 (1)	0	17 (11)	0	26 (9)	0	22 (6)
Cephalosporins and enzyme inhibitors, *x* (% of *n*)	3 (100)	158 (99)	22 (100)	138 (89)	37	272 (91)	17 (100)	377 (94)
Other antibiotics (J01X), *n* (% of *N*)	100 (18)	62 (6)	53 (3)	33 (2)	437 (16)	157 (13)	408 (28)	457 (27)
Imidazole derivatives (J01XD)**, *x* (% of *n*)	100 (100)	62 (97)	53 (100)	31 (94)	437 (100)	157 (100)	408 (100)	453 (99)
Other antibiotics (J01XX), *x* (% of *n*)	0	2 (3)	0	2 (6)	0	0	0	4 (1)

*EASI* emergency abdominal surgery indications, *LUTSI* lower urinary tract surgery indications, *N* total number of prescriptions in the diagnosis group, *n* number of prescriptions for the third-level ATC pharmacological sub-group, *RASI* routine abdominal surgery indications, *UUTSI* upper urinary tract surgery indications, *x* number of prescriptions for the fourth-level ATC pharmacological sub-group. Percentages were rounded and presented in parenthesis

^*^Fixed-dose combinations that were not assigned by WHOCC, a fourth level ATC-code

^**^The imidazole derivate group also included oral metronidazole categorized as P01AB01

The overall mortality was significantly higher in the TH than in the NTH (*p* < 0.001). In the TH, 39 patients died (1%), of those, 8 had perforation peritonitis, 6 had an intestinal obstruction, 6 had cancers, and 4 were burn cases, while in NTH, 6 inpatients died (0.2%) who had diagnoses other than selected groups.

## Discussion

To the best of our knowledge, this is the first study that presents the antibiotic prescribing patterns among inpatients having the surgical diagnosis from two Indian private sector hospitals. Irrespective of the presence or absence of infectious indication, almost all inpatients across the four most common surgical diagnoses groups (UUTSI, LUTSI, RASI, and EASI) were prescribed antibiotics. The universal recommendation to administer a single dose of antibiotic preoperatively, as prophylaxis, was not followed in any patents in both study settings. High proportions of broad-spectrum antibiotics were prescribed for a long duration in the settings. Antibiotics were prescribed with higher DDDs/100 patient days in the NTH than in the TH.

Nearly 90% of all inpatients at both settings were prescribed antibiotics during their hospital stay. The proportion of patients who were prescribed antibiotics in this study was higher than shown in a 5-month hospital-level study (79–82%) conducted previously at the study settings (Sharma et al. 2012). Thus, the result of the present study endorses the results of the global antibiotic consumption data from 2000 to 2010, which states a rapid increase in per capita antibiotic use in India (Van Boeckel et al. 2014). Escalation of infectious diseases and the use of antibiotics as preventive medicine were considered as the main reasons for this increase (Van Boeckel et al. 2014).

The diagnostic tools, such as microbiology culture and susceptibility test reports justify the antibiotic prescribing and suggest the effective class of antibiotics to prescribe. Although empirical therapy often needs to be started before the diagnostic results are obtained, narrowing of the antibiotic therapy based on the lab reports helps to slow down the development of ABR. Despite the presence of microbiology facilities in proximity in both hospitals, microbiology tests were not advised frequently in the study hospitals. Most of the antibiotic prescriptions were managed empirically throughout the hospital stay. The results highlight that the microbiology lab facilities were underused in the study settings, therefore resulting in a continuation of antibiotic treatment. In the four selected diagnoses groups, none of the patients had any microbiologically confirmed bacterial infection; therefore, they ought not to receive any antibiotic treatment but only a recommended dose of PAP.

Antibiotic prophylaxis is defined as the administration of a single dose of the effective antimicrobial agent prior to the exposure with possible contamination, i.e., surgery to reduce the risk of SSI (World Health Organization 2016). This single dose of antibiotic is recommended to be given 120 to 60 min before surgical incision. The most recent guidelines of the Centers for Disease Control and Prevention (CDC) restricts any additional prophylactic antibiotics after the completion of the clean and clean-contaminated surgical procedures (Crader and Bhimji 2018). Despite these recommendations, antibiotics were prescribed for a longer time period as a treatment regime to a majority of the inpatients in both settings.

Cefazolin (J01DB04), a first-generation cephalosporin, provides adequate coverage against most of the organisms causing postoperative infections; it causes minimal allergic reactions and side effects, achieves adequate tissue levels, and is relatively less expensive. The presence of these benefits makes cefazolin the most appropriate PAP agent for the majority of the surgical procedures (World Health Organization 2016). Overall, cefazolin was prescribed only in ≤ 28% cases in the TH and even significantly less in the NTH (≤ 5%, *p* < 0.05). In the EASI group, imidazole derivatives were most commonly prescribed in both hospitals. In the RASI group, aminoglycosides in the TH and FDCs of third-generation cephalosporins in the NTH were commonly prescribed instead of cefazolin (World Health Organization 2016). In both the UUTSI and the LUTSI groups, quinolones were the most commonly prescribed in both hospitals (Table 3). The administration of quinolones as prophylaxis is recommended for specific urogynecology surgical procedures that were not performed frequently in the study settings (Bulletins--Gynecology 2009). Therefore, the classes of antibiotics prescribed in the selected diagnoses groups could not be considered as optimal PAP treatment as per international guidelines and the prescription pattern might need a reconsideration.

An average antibiotic period for all four groups was almost equal to the average hospitalization duration for most of the patients in both hospitals specifically in the TH (Table 1). The hospital stay and duration of antibiotic treatment in the TH were significantly longer compared to the NTH. This can be due to the free service provision, including medicines and the diagnostics, to all patients in the TH. On the contrary in the NTH, patients had to pay out-of-pocket for provided services and medicines. Spending money out-of-pocket might be shorter length of stay and shorter duration of prescription in the NTH, the inpatients at the NTH. Although, prescribing antibiotics for shorter or longer time periods than recommended, is considered inappropriate being a trigger for the development and spread of ABR.

Similar to our study results, an Iranian study showed antibiotic treatment in 83% of cases though it was indicated only in 37% cases (Vessal et al. 2011). The duration of antibiotic administration was extended beyond the prophylactic period in a questionnaire survey for colorectal surgery at 721 Japanese institutions and analysis of 34133 medical records of patients undergoing 5 major surgical procedures in the USA (Kobayashi et al. 2011; Bratzler et al. 2005). Unindicated prescribing of antibiotics and its extension for long durations to prevent SSIs in high-risk patient groups is a global problem and a major contributing factor for the development of ABR (Bulletins--Gynecology 2009; Vessal et al. 2011). Dissemination of knowledge into practice is a challenging and long-term process (Vessal et al. 2011; Kobayashi et al. 2011; Bratzler et al. 2005). It also plays a pivotal role in increasing the cost of treatment, imposing a high economic burden on the country, patients, and healthcare systems, and increases resource constraints specifically in countries like India.

Our study also highlights the selection and prescribing of broad-spectrum antibiotics such as fluoroquinolones, FDCs of third-generation cephalosporins, imidazole derivatives, and aminoglycosides throughout the selected diagnoses groups regardless of the surgery indications. The recommended narrow-spectrum antibiotics such as penicillins were used to a limited extent in both hospitals which shows a clear deviation from the available international guidelines provided by the CDC (Kobayashi et al. 2011). The inpatients at the TH were more likely to receive tetracycline, lincosamides, quinolones, and imidazole derivatives, while inpatients at the NTH were more often prescribed FDCs of cephalosporins. Most of the prescribed FDCs of a cephalosporin and a beta-lactamase inhibitor (16–47% in the NTH) across all four diagnosis groups have no underlying scientific justification (Table 3). These combinations are justified only in case of ESBL-producing strains of *E.coli* and *Klebsiella.* This practice not only increases the treatment cost but also is a contributor to the development of ABR. In a single-center study in Andhra Pradesh, India, Khade et al*.* observed high use of fluoroquinolones (28%), cephalosporins (18%), aminoglycosides (14%), and imidazole derivatives (22%) in a surgery department (Khade et al. 2013). However, high prescribing of the FDCs in our settings is a point of concern.

Local prescribing guidelines are adjusted to the local disease panorama and resistance patterns; therefore, its implementation can improve antibiotic prescribing practices and minimize the development of ABR (Bulletins--Gynecology 2009; Vessal et al. 2011; CDC 2018; Khade et al. 2013; Parulekar et al. 2009). There is an urgent need to develop and implement the local prescribing guidelines, and prescription monitoring for justified use of antibiotics. Our results also recommend to explore the underlying reasons for such prescribing patterns using both qualitative and quantitative approaches among the prescribers, policymakers, community, and dispensers.

Other highlights of the study are significantly less prescribing of antibiotics by generic names and a higher proportion of antibiotics prescribed via the parenteral route of administration with higher defined daily doses (in DDDs/100 patient days) in the NTH compared to the TH; across all four diagnosis groups. The generic medicines are bioequivalent to the branded medicines, are cost-effective compared to branded medicines, and promise compliance with the prescription (Sharma et al. 2012). At the TH, the purchase and supply of the medicines are managed by the hospital management, which is obliged to maintain a good quality of medicines with the lowest possible costs. This might be the reason for higher generic name prescribing at the TH. Moreover, the prescribers at the NTH were exposed to the sales representatives of pharmaceutical companies but not at the TH. This exposure might have resulted in higher pressure on the prescribers of the NTH for prescribing medicines by brand names (Blumenthal 2004). Other possible explanations for a more conservative approach for the DDDs/100 patient days in the TH can be the updated knowledge about the severity of the problem of ABR through continued medical education programs. These programs are regularly conducted as a part of the curriculum at the TH, but not at the NTH. A strong customer-supplier-oriented relationship between the patient and the prescriber is developed when the patients pay out-of-pocket for the healthcare, as in the NTH. The patients in such situations demand quick relief through antibiotics, and prescribers aim to satisfy the patients’ demand. This was observed previously in studies from India and Malaysia (Kotwani et al. 2010; Ab Rahman et al. 2016). The assessment of demand-supply relation was not conducted in the present study being beyond the aim but could be a part of the future study.

## Strengths and limitations

A major strength of this study is the manual prospective data collection method among a large group of patients during 3 years’ time period in two private hospitals in India. In the present study, demographic characteristics were not considered as the selection parameter. This allowed the inclusion of a wide cross-section of the inpatients. The study provided a diagnose-specific detailed record of prescribing antibiotics in surgery departments in two private sector hospitals (a teaching and a non-teaching). The study population and their disease panorama were comparable with populations from other similar settings and may serve as representative of average Indian surgery departments. The data was collected for a long time period which helps to overcome the confounding factors caused by seasonal changes. In addition, this study gives an overview of antibiotic use and fills up the surveillance knowledge gap necessary to take further steps to combat antibiotic resistance. It is also in line with the Global Action Plan to Combat Antimicrobial Resistance.

Several limitations of this study need to be acknowledged. Firstly, the absence of computerized record systems in hospitals and of personal identification numbers, untrained staff, and high staff turnover make a detailed study like this time-consuming and onerous and delay the analysis. However, the use of manpower is the only option to conduct such detailed studies at resource-constrained settings but at the same time leads to a more accurate description of the prescribing patterns. The authors are aware that extensive manual checking and adding the ICD codes and ATCs for the new FDCs to the data have prolonged the analysis and delayed the presentation. Although, the data represented in this paper is from 2008 till 2011, however, similar trend for antibiotic use has been predicted through extrapolation in HISTI model by Tamhankar et al. (Tamhankar et al. 2018) which increases the validity of the result of our study also in present context. Moreover, the ATC codes of the new FDCs assigned by the authors will serve as a reference point for other studies to be published in the future. The situation we want to emphasize here is the high prescribing of FDCs, broad-spectrum antibiotics, and prescribing these for an incorrect indication for inappropriate time. Also, the lack of national PAP guidelines and local prescribing guidelines in hospitals is still present. We, therefore, believe that the results of the current study, as well as upcoming publications from these settings, will be a starting point for the discussion on the necessity of local prescribing guidelines and a driving force for action.

Finally, the diagnoses were not validated externally, as this was not the aim of the study. The detailed information regarding the surgeries or risk factors for surgical patients was not collected in this study. The results of this study demonstrate the need for context-specific guidelines to regulate the use of antibiotics during surgical procedures:

- Large patient group with no exclusion due to demographic variables
- Disease panorama similar to other Indian settings
- Data collection during a long time period decrease confounding by seasonal change
- No data on actual surgeries collected
- No data on antibiotic resistance patterns of pathogens in the setting

## Conclusion

This study highlights an extensive prescribing of antibiotics, specifically broad spectrum antibiotics including FDCs across the departments and in selected most common surgical diagnosis groups irrespective of any indication of an infection. The universal recommendations of preoperative use of antibiotics were not followed in majority of the cases in both hospitals. The study also highlights underuse of the microbiology diagnostic tools and extended empirical prescribing. Thus, the results clearly point towards an urgent need to develop and implement locally adjusted diagnose-specific antibiotic prescribing guidelines and regular monitoring thereafter at the settings. The results of the present study are expected to have a significant impact on the prescribers, to initiate the development of local prescribing guidelines.

## Future implications

The result of the study could be provided as feedback to the prescribers to encourage them to utilize the diagnostic facilities. This will help to conduct studies to describe the ABR pattern in pathogens in the region and then to evaluate the rationality of selection of prescribed antibiotics in order to create locally adjusted diagnosis-specific antibiotic prescribing guidelines. The ATC codes of the new FDCs assigned by the authors could be used as a reference for other studies for better comparison of the results. Qualitative studies are needed to understand the factors contributing to the present antibiotic prescribing patterns. In parallel, several educational interventions targeting community about not demanding antibiotics and not self–medicating with antibiotics are suggested.

## Data Availability

The data is with the institutional ethics committee as per the institutional policy. This is to protect the patient’s confidentiality and to ensure the electronic security of the data. The data will be made available to all interested researchers upon request made to the Chairman of the Ethics Committee, R.D. Gardi Medical College, Agar Road, Ujjain, Madhya Pradesh, India 456006 (email: iecrdgmc@yahoo.in, uctharc@bsnl.in), giving all details of the article. The ethical approval number: 41/ 2007 and 114/2010 needs to be quoted along with the request.

## Abbreviations

ABR: Antibiotic resistance
ATC: Anatomical Therapeutic Chemical
CDC: Centers for Disease Control and Prevention
DDD: Defined daily doses
EASI: Emergency abdominal surgery indications
FDCs: Fixed-dose combinations
HAIs: Healthcare-associated infections
HIC: High-income countries
ICD 10: International Statistical Classification of Diseases and Related Health Problems - Tenth Revision
LMIC: Low- and middle-income countries
LUTSI: Lower urinary tract surgery indications
NLEMI: National List of Essential Medicines of India
NTH: Non-teaching hospital
PAP: Perioperative antibiotic prophylaxis
RASI: Routine abdominal surgery indications
SSIs: Surgical site infections
TH: Teaching hospital
UTI: Urinary tract infection
UUTSI: Upper urinary tract surgery indications
WHO: World Health Organization
WHOLEM: WHO List of Essential Medicines
