# Correction to: Perioperative antibiotic prescribing in surgery departments of two private sector hospitals in Madhya Pradesh, India

**DOI:** 10.1186/s13741-019-0123-1

**Published:** 2019-10-28

**Authors:** Anna Machowska, Jonatan Sparrentoft, Shyam Kumar Dhakaita, Cecilia StålsbyLundborg, Megha Sharma

**Affiliations:** 1 Department of Public Health Sciences, Global Health - Health Systems and Policy, Karolinska Institutet, 17177 Stockholm, Sweden; 2 Department of Pharmacology, Ruxmaniben Deepchand Gardi Medical College, Surasa, Ujjain, 456006 India; 3 Department of Surgery, Ruxmaniben Deepchand Gardi Medical College, Surasa, Ujjain, 456006 India

**Correction to: Perioper Med (2019) 8:10**

**https://doi.org/10.1186/s13741-019-0121-3**

Following publication of the original article (Machowska et al. 2019), the authors provided new footnotes for Table 1 and Table 2. The complete tables and updated footnotes are supplied below.

**Table 1:** Inpatients’ characteristics at surgery departments of the TH and NTH in Madhya Pradesh, India

	All patients		UUTSI		LUTSI		RASI		EASI	
	TH,	NTH,	TH,	NTH,	TH,	NTH,	TH,	NTH,	TH,	NTH,
	*N* = 6171	*N* = 6263	*N* = 284	*N* = 741	*N* = 748	*N* = 824	*N* = 1394	*N* = 748	*N* = 482	*N* = 763
	*n* (%)	*n* (%)	*n* (%)	*n* (%)	*n* (%)	*n* (%)	*n* (%)	*n* (%)	*n* (%)	*n* (%)
Age in years										
0–17	*677 (11)* ^a^	377 (6)	21 (7)	36 (5)	*45 (6)* ^a^	15 (2)	*139 (10)* ^a^	26 (3)	72 (15)	91 (12)
18–39	2018 (33)	*2757 (44)* ^a^	159 (56)	425 (57)	69 (9)	119 (14)	320 (23)	*281 (38)* ^a^	239 (50)	419 (55)
40–59	1773 (29)	1689 (27)	78 (27)	197 (27)	150 (20)	148 (18)	453 (32)	222 (30)	108 (22)	188 (25)
≥ 60	*1703 (28)* ^a^	1440 (23)	26 (9)	83 (11)	484 (65)	542 (66)	482 (35)	219 (29)	63 (13)	65 (8)
Male	*4601 (75)* ^a^	4384 (70)	218 (77)	546 (74)	719 (96)	812 (99)	*1182 (85)* ^a^	532 (71)	347 (72)	610 (80)
Female	1570 (25)	*1879 (30)* ^a^	66 (23)	195 (26)	29 (4)	12 (1)	212 (15)	*216 (29)* ^a^	135 (28)	153 (20)
Patients prescribed AB	5430 (88)	5402 (86)	251 (88)	624 (84)	748 (100)	823 (100)	*1393 (100)* ^a^	628 (84)	*482 (100)* ^a^	719 (94)
Total number of AB prescriptions (Np)	12,213	9598	563	1055	1624	1453	2767	1240	1435	1679
Prescriptions adherent to WHOLEM, Np (percentage of total nr. of prescriptions)	*8039 (66)* ^a^	4019 (42)	*309 (55)* ^a^	346 (33)	*753 (46)* ^a^	346 (24)	*1736 (63)* ^a^	510 (41)	*1054 (73)* ^a^	790 (47)
Prescriptions adherent to NLEMI, Np (percentage of total nr. of prescriptions)	*9119 (75)* ^a^	5487 (57)	342 (61)	592 (56)	*880 (54)* ^a^	592 (41)	*2025 (73)* ^a^	734 (59)	*1173 (82)* ^a^	984 (59)
Average length of hospital stay, days [SD]	*9.9 [10.0]* ^a^	4.6 [3.9]	*9.1 [7.8]* ^a^	3.8 [3.2]	*13.0 [11.3]* ^a^	5.0 [3.2]	*9.8 [7.2]* ^a^	5.1 [4.0]	*11.1 [9.5]* ^a^	6.3 [5.3]
Average length of AB treatment, days [SD]	*9.5 [7.6]* ^a^	4.4 [3.2]	*8.6 [6.9]* ^a^	3.6 [2.6]	*11.5 [9.0]* ^a^	4.9 [3.0]	*7.8 [5.3]* ^a^	4.4 [3.4]	*9.0 [7.4]* ^a^	5.5 [4.0]
AB prescribed for 1 day	36 (1)	221 (7)	7 (2)	96 (13)	6 (1)	44 (5)	14 (1)	41 (5)	9 (2)	40 (5)
DDD/100 patient days	72.5	*110.5* ^a^	89.5	*112.3* ^a^	84.6	*102.1* ^a^	78.3	*116.1* ^a^	74.9	*106.0* ^a^
Total nr. of AB doses administered	65,288	28,600	2759	2950	9415	4733	13,890	4060	6270	7980
Total nr. of AB doses prescribed using generic names	*16,322 (25)* ^a^	1716 (6)	*607 (22)* ^a^	118 (4)	*2542 (27)* ^a^	142 (3)	*4584 (33)* ^a^	203 (5)	*1693 (27)* ^a^	399 (5)
Total nr. of AB doses administered parenterally	34,613 (53)	*19,639 (79)* ^a^	1335 (41)	*2245 (76)* ^a^	3672 (39)	*2603 (55)* ^a^	8195 (59)	*3370 (83)* ^a^	4582 (75)	*7339 (92)* ^a^

Percentages were rounded off to integers, and standard deviations were rounded off to one decimal. Percentages are presented in parenthesis and standard deviations in square brackets. Significantly larger values are presented in italics

*Abbreviations*: *AB* antibiotic, *DDD* defined daily dose, *EASI* emergency abdominal surgery indications, *Np* total number of antibiotic prescriptions, *nr.* number, *LUTSI* lower urinary tract surgery indications, *NLEMI* National List of Essential Medicines of India, *NTH* non-teaching hospital, *RASI* routine abdominal surgery indications, *SD* standard deviation, *TH* teaching hospital, *UUTSI* upper urinary tract surgery indications, *WHOLEM* WHO List of Essential Medicines

^a^ Significant *p*-values

**Table 2:** Classification of surgery inpatients in four selected diagnoses groups, based on their diagnoses

Diagnosis groups (ICD-10 codes)	TH, *n* = 2908 (100) *n* (%) [%]	NTH, *n* = 3076 (100) *n* (%) [%]
Upper urinary tract surgery indications (UUTSI)	284 (10) [100]	*741 (24) [100]* ^a^
Kidney/ureter calculi (N20)	280 [99]	*734 [99]* ^a^
Other	4 [32]	7 [1]
Lower urinary tract surgery indications (LUTSI)	748 (26) [100]	824 (27) [100]
Benign prostate hyperplasia (N40)	522 [70]	558 [68]
Urethral/bladder calculi (N21)	89 [12]	*142 [17]* ^a^
Urethral stricture (N35)	85 [11]	66 [8]
Prostate cancer (C61)	29 [4]	30 [4]
Bladder cancer (C67)	10 [1]	10 [1]
Urethral disease^*^ (N36)	9 [1]	10 [1]
Other	4 [< 1]	8 [< 1]
Routine abdominal surgery indications (RASI)	*1394 (48) [100]* ^a^	748 (24) [100]
Hernia (K40-K46)	*965 [69]* ^a^	312 [42]
Other gastric/duodenal disease (K31)	113 [8]	151 [20]
Biliary calculi (K80)	73 [5]	110 [15]
Anal fissure/fistula (K60)	*103 [7]* ^a^	30 [4]
Malignancy in GI-tract (C15-C26)	78 [6]	51 [7]
Cholecystitis (K81)	30 [2]	43 [6]
Anal abscess (K61)	10 [< 1]	28 [4]
Other disease in anal region^**^ (K62)	10 [< 1]	8 [1]
Colon polyposis (K63.2)	9 [< 1]	5 [< 1]
Other	3 [< 1]	10 [< 1]
Emergency abdominal surgery indications (EASI)	482 (17) [100]	*763 (25) [100]* ^a^
Appendicitis (K35-K37)	268 [56]	*459 [60]* ^a^
Intestinal obstruction (K56)	132 [27]	202 [27]
Peritonitis (K56)	80 [17]	99 [13]
Other	2 [< 1]	3 [< 1]

Numbers of patients with percentages rounded off to the nearest whole number

*Abbreviations: TH* teaching hospital, *NTH* non-teaching hospital

^*^Urethral disease includes fistulas, diverticulosis, carbuncles, and prolepses of the urethra

^**^Disease in the anal/rectal region includes polyps, stenosis, wounds, bleedings, and prolapses in the anal/rectal region

^a^ Significant *p*-values

## References

[CR1] Machowska (2019). Perioperative antibiotic prescribing in surgery departments of two private sector hospitals in Madhya Pradesh, India. Perioper Med.

